# Analysis of *MUC6* polymorphisms on the clinicopathologic characteristics of Asian patients with oral squamous cell carcinoma

**DOI:** 10.1111/jcmm.17886

**Published:** 2023-08-15

**Authors:** Chun‐Hung Hua, Chun‐Yi Chuang, Yi‐Chung Chien, Chun‐Wen Su, Shuo‐Chueh Chen, Liang‐Chih Liu, Shun‐Fa Yang, Yung‐Luen Yu

**Affiliations:** ^1^ Department of Otorhinolaryngology Head and Neck Surgery China Medical University Hospital Taichung Taiwan; ^2^ School of Medicine Chung Shan Medical University Taichung Taiwan; ^3^ Department of Otolaryngology Chung Shan Medical University Hospital Taichung Taiwan; ^4^ Graduate Institute of Biomedical Sciences China Medical University Taichung Taiwan; ^5^ Institute of Translational Medicine and New Drug Development China Medical University Taichung Taiwan; ^6^ Center for Molecular Medicine China Medical University Hospital Taichung Taiwan; ^7^ Institute of Medicine Chung Shan Medical University Taichung Taiwan; ^8^ Department of Medical Research Chung Shan Medical University Hospital Taichung Taiwan; ^9^ Division of Pulmonary and Critical Care Medicine, Department of Internal Medicine China Medical University Hospital Taichung Taiwan; ^10^ School of Medicine, College of Medicine China Medical University Taichung Taiwan; ^11^ Department of Surgery China Medical University Hospital Taichung Taiwan; ^12^ Department of Medical Laboratory Science and Biotechnology Asia University Taichung Taiwan

**Keywords:** lymph node metastasis, *MUC6*, oral squamous cell carcinoma, single‐nucleotide polymorphisms

## Abstract

Head and neck squamous cell carcinomas (HNSCCs) are generally associated with tobacco consumption, alcohol abuse or both. Mucins (MUCs) are high‐molecular‐weight glycoproteins produced by many epithelial tissues. Many studies have indicated that MUCs play an important role in cancer metastasis. *MUC6* expression has been observed in gastric and oncocytic phenotypes and plays an important role during cancer progression. We found that levels of *MUC6* are lower in Asian HNCC patients and affect the disease‐free survival of HNCC patients. Next, we investigated the combined effect of *MUC6* polymorphisms and exposure to environmental carcinogens on the susceptibility to and clinicopathological characteristics of HNCC. Three single‐nucleotide polymorphisms (SNPs) of *MUC6* (rs7481521, rs6597947 and rs61869016) were analysed using real‐time PCR. After adjusting for other co‐variants, we found that carrying a CC genotype at *MUC6* rs6597947 led to a lower risk of developing oral squamous cell carcinoma (OSCC) than wild‐type carriers among non‐betel‐quid chewers. Moreover, male oral cancer patients who carried the AA + CC genotype at *MUC6* rs6597947 had a lower risk of lymph node metastasis than other genotypes, suggesting a significant functional compromise and decompensated disease. Therefore, our findings suggest that genetic variations in *MUC6* may correlate to OSCC and indicate the progression in OSCC patients.

## 1 INTRODUCTION

Head and neck squamous cell carcinomas (HNSCCs) are a group of biologically similar cancers originating in the oral cavity, nasopharynx, oropharynx, hypopharynx and larynx, which histologically present, mainly, as squamous cell carcinomas. HNSCC is the sixth most common malignancy worldwide, and one of the most common types of HNSCC is oral squamous cell carcinoma (OSCC).^1^, ^2^, ^3^, ^4^ Despite significant advances in combined multidisciplinary diagnoses and treatments, only 30%–50% of HNCC patients worldwide survive more than 5 years after initial diagnosis.^5^ This poor prognosis is due to the occurrence of distant metastases and local recurrence.^6^ OSCC occurs as a result of various genetic changes due to long‐term exposure to environmental carcinogens. The main risk factors for OSCC are chronic inflammation, tobacco use, alcohol consumption, betel‐nut chewing and viral infection. Mucin (MUC) is the main component of any mucus secretion, which provides the mucus with its biophysiochemical properties as a function of its characteristics and a degree of glycosylation.^7^, ^8^ Moreover, mucins play a role in both physiological and pathological conditions.^9^, ^10^, ^11^, ^12^, ^13^ Aberrant expression of mucins can lead to a loss in epithelial cell polarity and promote epithelial–mesenchymal transition (EMT), leading to increased cell motility and invasion, a crucial step in tumorigenesis.^9^, ^14^, ^15^

Reports suggest that MUC1 modulates the impact of hypoxia in head and neck squamous cell carcinoma (HNSCC) cells by regulating HIF‐1α.^16^ MUCs can be found either membrane‐bound or secreted with or without gel formation. Among the membrane‐bound MUCs, MUC1, 4, 15 and 16 have received the most attention, whereas MUC2, 5B, 5AC, 6 and 19 have received the most attention among the gel‐forming MUCs.^9^, ^17^, ^18^ Over the last decade, numerous independent works have evaluated the clinical significance of MUC protein expression in cancer as well as its ability to predict outcomes.^19^, ^20^, ^21^, ^22^ Despite these efforts, the findings from these studies have been inconsistent and no definitive conclusion has yet been reached. One of the primary elements of the mucus barrier in the stomach is Mucin 6 (MUC6), which is produced by the pyloric gland cells of the gastric sinus and the mucus neck cells in the lower layer of the gastric mucosa. Both gastric and cancer cell types exhibit *MUC6* expression. In our previous study, we found that genetic variations in *MUC6* may correlate to hepatocellular carcinoma and indicate the progression in hepatocellular carcinoma patients.^23^ Research has revealed that the methylation of the *MUC6* promoter may cause a considerable decrease in *MUC6* expression in gastric cancer and drive its progression.^21^ Furthermore, we found low *MUC6* expression reduced the risk of OSCC. However, the detailed role of the tissue expression of mucins in OSCC tumour cells is not well understood.

Studies have revealed several genetic predisposition factors that could play a role in HNSCC. One of the most prevalent forms of DNA sequence variations is single‐nucleotide polymorphisms (SNPs), which have been demonstrated to have the capability to predict the probability of developing cancer.^24^, ^25^, ^26^ SNPs can change the expression or function of proteins, affecting the development of cancer. A connection was discovered between the expression of *MUC6* SNPs and the onset of chronic atrophic gastritis and hepatocellular carcinoma.^27^ However, the exact role of *MUC6* SNPs in cancer progression and development in Taiwanese OSCC patients remains poorly investigated. In addition, there is a strong correlation between the incidence of oral squamous cell carcinoma and gender in Taiwan. The occurrence ratio between males and females is approximately 9:1, which may be related to the fact that the majority of betel‐nut chewers are male. Therefore, the current study aims to investigate the correlations between three *MUC6* SNPs (rs61869016, located in the 5′‐UTR; rs6597947, located in the 5′‐UTR and rs7481521, located in the exon) and Taiwanese male patients with OSCC and their cancer prognosis.

## 2 MATERIALS AND METHODS

### 2.1 Study participants and specimen collection

In this research, 1115 male patients diagnosed with oral squamous cell carcinoma (OSCC) were selected from Chung Shan Medical University Hospital located in Taichung, Taiwan. The participants were required to give written consent after being fully informed about the study. At the time of diagnosis, the OSCC patients were assigned a clinical stage based on the American Joint Committee on Cancer's tumour/node/metastasis staging system (AJCC, 2002). For the control group, 837 individuals, aged between 20 and 70 years with no history of cancer, were selected from the Taiwan Biobank (https://www.twbiobank.org.tw/new_web_en/index.php).

Data on gender and age, as well as habits of betel‐quid chewing, cigarette smoking and alcohol consumption, were gathered from each participant. The consumption of more than two alcoholic beverages per day by a subject was classified as alcohol consumption. If an individual smoked one cigarette per day in the past 3 months, they were considered to have a chronic smoking habit. The study received approval from the Institutional Review Board at Chung Shan Medical University Hospital.

### 2.2 Comprehensive analyses of MUC6 from The Cancer Genome Atlas (TCGA)

UALCAN is a comprehensive, user‐friendly and interactive web resource for analysing cancer omics data (http://ualcan.path.uab.edu/index.html). UALCAN uses TCGA level 3 RNA‐seq and clinical data from 31 cancer types.^28^ Gene Expression Profile Interactive Analysis 2 (GEPIA2, http://gepia2.cancer‐pku.cn/#index) is an updated version of GEPIA, used to analyse the RNA‐sequencing expression data of 9736 tumours and 8587 normal samples from the TCGA and the GTEx project using a standard processing pipeline.^29^ In this study, we used UALCAN and GEPIA2 for analyses of tumour/normal differential expression and overall survival of *MUC6* expression in HNSCC patients.

### 2.3 Selection of MUC6 polymorphisms

For this study, three SNPs in *MUC6* (NM_005961.3) were selected from the International HapMap Project data. These SNPs were rs61869016, located in the 5′‐UTR; rs6597947, located in the 5′‐UTR and rs7481521, located in the exon of *MUC6*.

### 2.4 MUC6 genotyping

The allelic discrimination of *MUC6* polymorphisms rs61869016, rs6597947 and rs7481521 was assessed using an ABI StepOne real‐time polymerase chain reaction system (Applied Biosystems), SDS v3.0 software (Applied Biosystems) and the TaqMan assay.^24^

### 2.5 Statistical analyses

To evaluate the differences in age and demographic characteristics between the control group and the OSCC patients, the Mann–Whitney *U* test was used. The odds ratios with 95% confidence intervals (CIs) were estimated using logistic regression models. A *p* value of <0.05 was considered significant. The data were analysed using SAS statistical software.

## 3 RESULTS

**FIGURE 1:**
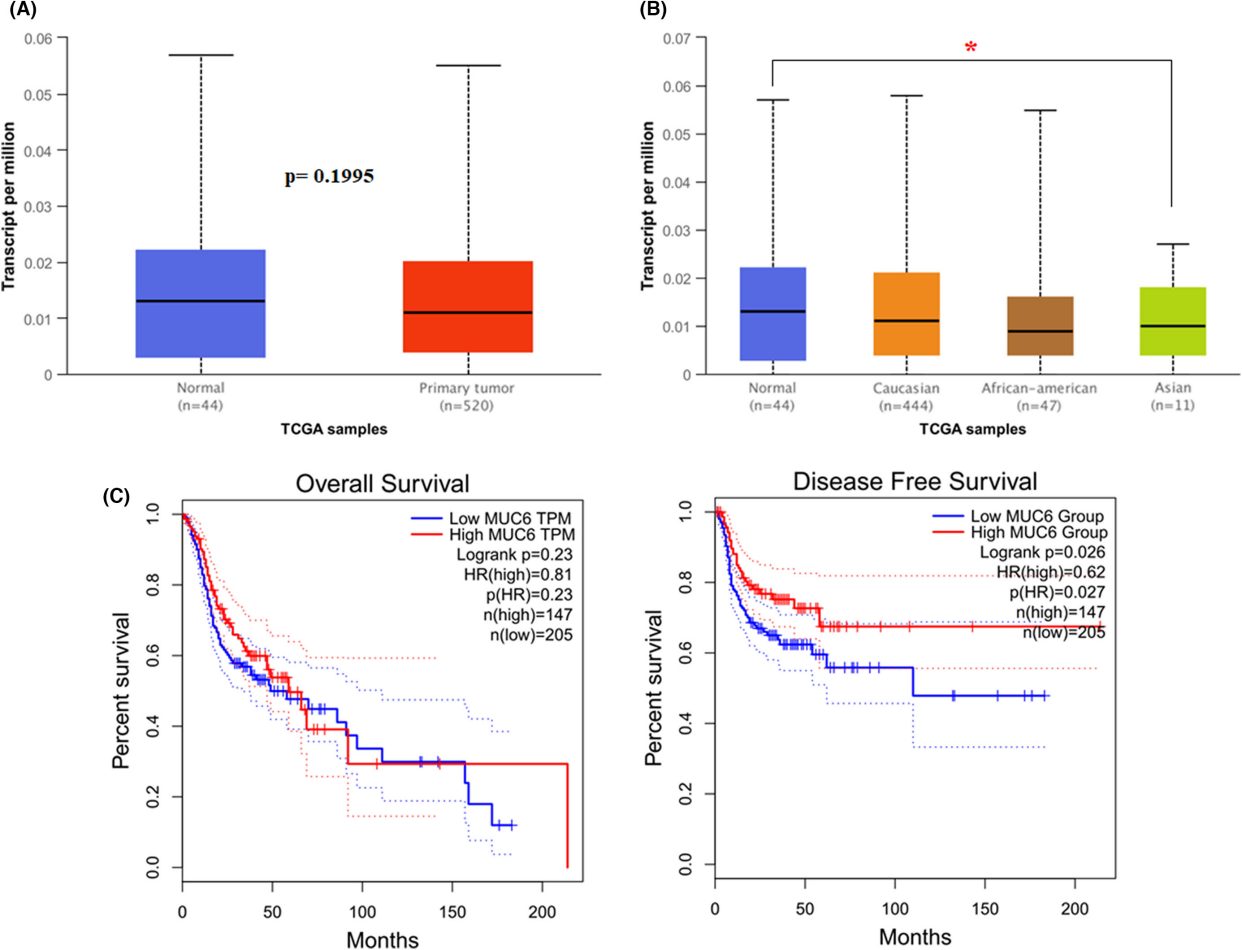
The level of *MUC6* is correlated with Asian HNSCC progression and with the disease‐free survival rate of HNSCC. (A) The level of *MUC6* in control and HNSCC patients. (B) The level of *MUC6* in control and HNSCC patients of different human races. (C) The overall survival and disease‐free survival of different levels of *MUC6* in HNSCC patients as assessed with data from GEPIA2. *p < 0.05.

To investigate the clinical impact of *MUC6* on HNSCC progression, we used UALCAN and GEPIA 2 to assess the relationship between the cellular levels of *MUC6* of the controls and the HNSCC patients and the overall or disease‐free survival of the HNSCC patients. Initially, however, there was no difference in *MUC6* levels between the controls and the HNSCC patients (*p* = 0.1995; Figure 1A). Upon further analyses, we found that levels of *MUC6* in the controls were significantly higher than in Asian HNSCC patients (*p* = 0.0499; Figure 1B). Interestingly, it was found that expression of *MUC6* affects the disease‐free survival of HNSCC patients. The HNSCC patients with high MUC6 expression had significantly longer disease‐free survival than those with low MUC6 expression (Figure 1C). These results imply that the regulation of *MUC6* might be involved in HNSCC progression.

To identify possible factors causing OSCC in clinical practice, a total of 1115 healthy controls and 837 male OSCC patients were recruited for this case‐cohort study. According to our analyses of OSCC patients, we found significant differences in age (*p* < 0.001), betel‐nut chewing (*p* < 0.001), smoking (*p* < 0.001) and alcohol consumption (*p* < 0.001) between the OSCC patients and the healthy group (Table 1).

**TABLE 1:** The distributions of demographical characteristics in 837 controls and 1115 male patients with oral cancer.

Variable	Controls (*N* = 837)	Patients (*N* = 1115)	*p* value
	Mean ± SD	Mean ± SD	
Age (years)	59.51 ± 7.06	55.63 ± 10.87	<0.001*
Betel‐quid chewing			
No	719 (85.9%)	291 (26.1%)	
Yes	118 (14.1%)	824 (73.9%)	<0.001*
Cigarette smoking			
No	393 (47.0%)	183 (16.4%)	
Yes	444 (53.0%)	932 (83.6%)	<0.001*
Alcohol drinking			
No	678 (81.0%)	606 (54.3%)	
Yes	159 (19.0%)	509 (45.7%)	<0.001*
Stage			
I + II		534 (47.9%)	
III + IV		581 (52.1%)	
Tumour T status			
T1 + T2		546 (49.0%)	
T3 + T4		569 (51.0%)	
Lymph node status			
N0		741 (66.5%)	
N1 + N2 + N3		374 (33.5%)	
Metastasis			
M0		1106 (99.2%)	
M1		9 (0.8%)	
Cell differentiation			
Well differentiated		164 (14.7%)	
Moderately or poorly differentiated		951 (85.3%)	

*Note*: Mann–Whitney *U* test or Fisher's exact test was used between healthy controls and patients with oral cancer.

* *p* value <0.05 was considered statistically significant.

To reduce the possibility of confounding by several environmental factors, AORs and their corresponding 95% CIs were estimated by multivariate logistic regression models after controlling for age, betel‐quid chewing, cigarette smoking and alcohol drinking. The genotype distributions and the associations between OSCC and *MUC6* SNPs are presented in Table 2. The alleles with the highest frequency of distribution in *MUC6* rs7481521, rs6597947 and rs61869016 were homozygous C/C, homozygous A/A and homozygous T/T, respectively, in the OSCC patients and the controls. However, individuals with the rs7481521, rs6597947 and rs61869016 polymorphisms showed no reduction in OSCC risk compared to the wild‐type individuals.

**TABLE 2:** Odds ratios (ORs) and 95% confidence intervals (CIs) of oral cancer associated with *MUC6* genotypic frequencies.

Variable	Controls (*N* = 837) (%)	Patients (*N* = 1115) (%)	OR (95% CI)	*p*	AOR (95% CI)^a^	*p*
rs7481521						
CC	425 (50.8)	555 (49.8)	1.000 (reference)		1.000 (reference)	
CT	332 (39.7)	449 (40.3)	1.036 (0.856–1.252)	0.718	1.010 (0.796–1.282)	0.933
TT	80 (9.5)	111 (9.9)	1.062 (0.776–1.454)	0.706	0.943 (0.635–1.400)	0.771
CT + TT	412 (49.2)	560 (50.2)	1.041 (0.870–1.245)	0.662	0.997 (0.796–1.249)	0.978
rs6597947						
AA	456 (54.5)	602 (54.0)	1.000 (reference)		1.000 (reference)	
AC	317 (37.9)	436 (39.1)	1.042 (0.862–1.259)	0.671	1.051 (0.830–1.332)	0.678
CC	64 (7.6)	77 (6.9)	0.911 (0.640–1.297)	0.606	0.685 (0.435–1.077)	0.102
AC + CC	381 (45.5)	513 (46.0)	1.020 (0.852–1.221)	0.830	0.985 (0.786–1.234)	0.895
rs61869016						
TT	342 (40.9)	437 (39.2)	1.000 (reference)		1.000 (reference)	
TC	375 (44.8)	514 (46.1)	1.073 (0.883–1.303)	0.479	1.080 (0.846–1.378)	0.535
CC	120 (14.3)	164 (14.7)	1.070 (0.813–1.408)	0.631	1.044 (0.738–1.475)	0.809
TC + CC	495 (59.1)	678 (60.8)	1.072 (0.893–1.287)	0.456	1.071 (0.851–1.348)	0.557

*Note*: The odds ratio (OR) with their 95% confidence intervals were estimated using logistic regression models.

^a^ The AOR with their 95% confidence intervals were estimated using multiple logistic regression models after controlling for age, betel‐quid chewing, cigarette smoking and alcohol drinking.

According to recent research, betel‐nut chewing is an important risk factor for OSCC progression. Therefore, genotyping and allele frequency data for *MUC6* SNPs among betel‐quid chewers are shown in Table 3. Unfortunately, the results showed no reduction in OSCC risk compared to the wild type among betel‐quid chewers. Because the risk factor of betel‐nut chewing is so influential, we removed this risk for further analyses. Surprisingly, the results showed that patients with the C/C genotype of rs6597947 SNP (OR = 0.394, 95% CI: 0.194–0.816, *p* = 0.012) had a reduced risk of OSCC compared to other genotypes among non‐betel‐quid chewers (Table 4).

**TABLE 3:** Odds ratios (ORs) and 95% confidence interval (CIs) of oral cancer associated with *MUC6* genotypic frequencies among betel‐quid chewers.

Variable	Controls (*N* = 118) (%)	Patients (*N* = 824) (%)	OR (95% CI)	*p*	AOR (95% CI)^a^	*p*
rs7481521						
CC	63 (53.4)	407 (49.4)	1.000 (reference)		1.000 (reference)	
CT	44 (37.3)	330 (40.0)	1.161 (0.769–1.752)	0.477	1.150 (0.758–1.743)	0.511
TT	11 (9.3)	87 (10.6)	1.224 (0.620–2.419)	0.560	1.195 (0.601–2.375)	0.611
CT + TT	55 (46.6)	417 (50.6)	1.174 (0.797–1.728)	0.417	1.159 (0.784–1.712)	0.459
rs6597947						
AA	66 (55.9)	440 (53.4)	1.000 (reference)		1.000 (reference)	
AC	43 (37.9)	316 (38.4)	1.102 (0.731–1.662)	0.642	1.092 (0.722–1.652)	0.677
CC	9 (7.6)	68 (8.2)	1.133 (0.540–2.380)	0.741	1.204 (0.568–2.553)	0.627
AC + CC	52 (44.1)	384 (46.6)	1.108 (0.751–1.663)	0.606	1.111 (0.751–1.645)	0.598
rs61869016						
TT	52 (44.1)	324 (39.3)	1.000 (reference)		1.000 (reference)	
TC	52 (44.1)	375 (45.5)	1.157 (0.766–1.748)	0.487	1.146 (0.755–1.739)	0.522
CC	14 (11.9)	125 (15.2)	1.433 (0.767–2.677)	0.260	1.328 (0.707–2.495)	0.378
TC + CC	66 (55.9)	500 (60.7)	1.216 (0.824–1.795)	0.325	1.185 (0.799–1.757)	0.398

*Note*: The odds ratios (ORs) with their 95% confidence intervals were estimated using logistic regression models.

^a^ The AORs with their 95% confidence intervals were estimated using multiple logistic regression models after controlling for age, cigarette smoking and alcohol drinking.

**TABLE 4:** Odds ratios (ORs) and 95% confidence intervals (CIs) of oral cancer associated with *MUC6* genotypic frequencies among non‐betel‐quid chewers.

Variable	Controls (*N* = 719) (%)	Patients (*N* = 291) (%)	OR (95% CI)	*p*	AOR (95% CI)^a^	*p*
rs7481521						
CC	362 (50.4)	148 (50.9)	1.000 (reference)		1.000 (reference)	
CT	288 (40.0)	119 (40.9)	1.011 (0.759–1.346)	0.942	0.954 (0.713–1.276)	0.749
TT	69 (9.6)	24 (8.2)	0.851 (0.515–1.406)	0.528	0.817 (0.492–1.356)	0.434
CT + TT	357 (49.6)	143 (49.1)	0.980 (0.746–1.286)	0.883	0.927 (0.703–1.222)	0.592
rs6597947						
AA	390 (54.2)	162 (55.7)	1.000 (reference)		1.000 (reference)	
AC	274 (38.1)	120 (41.2)	1.054 (0.795–1.398)	0.713	1.025 (0.770–1.364)	0.866
CC	55 (7.7)	9 (3.1)	0.394 (0.190–0.816)	**0.012** *	0.400 (0.192–0.833)	**0.014** *
AC + CC	329 (45.8)	129 (44.3)	0.944 (0.718–1.241)	0.680	0.923 (0.699–1.218)	0.570
rs61869016						
TT	290 (40.3)	113 (38.8)	1.000 (reference)		1.000 (reference)	
TC	323 (44.9)	139 (47.8)	1.104 (0.823–1.483)	0.509	1.057 (0.785–1.425)	0.714
CC	106 (14.8)	39 (13.4)	0.944 (0.616–1.447)	0.792	0.924 (0.601–1.421)	0.718
TC + CC	429 (59.7)	178 (61.2)	1.065 (0.806–1.407)	0.659	1.025 (0.773–1.359)	0.866

*Note*: The odds ratios (ORs) with their 95% confidence intervals were estimated using logistic regression models.

^a^ The AORs with their 95% confidence intervals were estimated using multiple logistic regression models after controlling for age, cigarette smoking and alcohol drinking.

* *p* value <0.05 was considered statistically significant in Bold.

The ORs analysed by their 95% CIs were estimated using logistic regression models: >T2, multiple tumours of more than 5 cm or a tumour involving a major branch of the portal or hepatic vein(s); *p < 0.05 was considered statistically significant.

Moreover, we further compared the associations between the *MUC6* rs6597947 polymorphism and the clinical status of OSCC patients to see how they related to the progression of clinical status in OSCC patients. The homozygous genotype for the polymorphic allele of rs6597947 (AC + CC) had a significantly lower‐level lymph node metastasis compared to the AA genotype in patients with OSCC (Table 5).

**TABLE 5:** Odds ratios (ORs) and 95% confidence intervals (CIs) of clinical statuses associated with genotypic frequencies of *MUC6* rs6597947 in male oral cancer patients.

Variable	Total (*N* = 1115)			Betel‐quid chewers (*N* = 824)			Non‐betel‐quid chewers (*N* = 291)		
	AA (*N* = 602)	AC + CC (*N* = 513)	*p* Value	AA (*N* = 440)	AC + CC (*N* = 384)	*p* Value	AA (*N* = 162)	AC + CC (*N* = 129)	*p* Value
Clinical stage									
Stage I + II	274 (45.5%)	260 (50.7%)	0.080	208 (47.3%)	196 (51.0%)	0.278	66 (40.7%)	64 (49.6%)	0.085
Stage III + IV	328 (54.5%)	253 (49.3%)		232 (52.7%)	188 (49.0%)		96 (59.3%)	65 (50.4%)	
Tumour size									
≦T2	297 (49.3%)	249 (48.5%)	0.740	223 (50.7%)	191 (49.7%)	0.794	74 (45.7%)	58 (45.0%)	0.879
>T2	305 (50.7%)	264 (51.5%)		217 (49.3%)	193 (50.3%)		88 (54.3%)	71 (55.0%)	
Lymph node metastasis									
No	382 (63.5%)	359 (70.0%)	**0.020** ^a^	286 (65.0%)	271 (70.6%)	0.089	96 (59.3%)	88 (68.2%)	0.081
Yes	220 (36.5%)	154 (30.0%)		154 (35.0%)	113 (29.4%)		66 (40.7%)	41 (31.8%)	
Metastasis									
M0	596 (99.0%)	510 (99.4%)	0.462	437 (99.3%)	381 (99.2%)	0.863	159 (98.2%)	129 (100.0%)	‐
M1	6 (1.0%)	3 (0.6%)		3 (0.7%)	3 (0.8%)		3 (1.8%)	0 (0.0%)	
Cell‐differentiated grade									
Well	87 (14.5%)	77 (15.0%)	0.830	74 (16.8%)	60 (15.6%)	0.644	13 (8.0%)	17 (13.2%)	0.143
Moderate or poor	515 (85.5%)	436 (85.0%)		366 (83.2%)	324 (84.4%)		149 (92.0%)	112 (86.8%)	

*Note*: Cell‐differentiated grade: grade I, well differentiated; grade II, moderately differentiated; grade III, poorly differentiated.

^a^ The adjusted odds ratios (AORs) with their 95% confidence intervals were estimated using multiple logistic regression models after controlling for age, betel‐quid chewing, cigarette smoking and alcohol drinking. AOR = 0.740 (0.575–0.953).

* *p* value <0.05 was considered statistically significant in Bold.

## 4 DISCUSSION

SNPs, or single‐nucleotide polymorphisms, are variations at the DNA level found in each human cell. These variations, influenced by environmental factors, contribute to the diversity of human phenotypes and can indicate a person's susceptibility to various diseases, including cancer.^30^, ^31^ To distinguish the impact of SNPs from other types of mutations, the frequency of each polymorphism must be higher than that of a single natural mutation. Changes in gene function caused by mutations or gene polymorphisms can result in increased cancer risk and specific disease phenotypes.^32^, ^33^

The laboratory evidence shows that an abnormal increase in the expression of tumour‐associated MUCs, mismatched features and shortened glycans is a common occurrence in various epithelial cancers.^34^, ^35^, ^36^, ^37^, ^38^ However, clinical findings are not always consistent. While most research suggests that high MUC levels are associated with poorer clinicopathological parameters and a worse prognosis, there are some studies that have reached the opposite conclusion.^39^, ^40^, ^41^, ^42^ The role of MUC1 in the EMT process, which enables cancer cells to become invasive and metastatic, has been directly proven by several studies. The importance of MUC6 in cancer is well known, but its precise role in tumorigenesis remains disputed as both oncogenic and inhibitory effects have been demonstrated.^19^, ^21^, ^43^ For example, the SNP rs7481521 of the MUC6 gene showed a significant decrease in risk for homozygous carriers and a clear relationship between the number of alleles and chronic atrophic gastritis.^27^ Moreover, *MUC6* expression is high in the early stages of pancreatic tumour progression, but it decreases or is lost in later stages.^44^, ^45^ However, the connection between MUC6 variations and risk factors for OSCC is still unclear. This study provides clarification on the role of MUC6 SNPs in OSCC susceptibility and other related conditions.

Taiwan has a higher incidence of head and neck cancer due to betel‐nut chewing and diet. Surprisingly, in our results, we observed that *MUC6* rs6597947 (AC + CC genotype) had a significantly lower‐level lymph node metastasis compared to the AA genotype in patients with OSCC (Table 5). As shown in Table 4, patients with the C/C genotype of the rs6597947 SNP had a reduced risk of OSCC compared to other genotypes among non‐betel‐quid chewers. Our findings suggest that genetic variations in MUC6 may help to predict cancer susceptibility in OSCC. This study provides new information about the relationship between MUC6 polymorphisms and the clinical pathology of OSCC in the Taiwanese population. However, the detailed mechanisms of *MUC6* SNPs in OSCC require future elucidation.

## AUTHOR CONTRIBUTIONS

**Chun‐Hung Hua:** Conceptualization (equal); writing – original draft (equal); writing – review and editing (equal). **Chun‐Yi Chuang:** Writing – original draft (equal). **Yi‐Chung Chien:** Methodology (equal). **Chun‐Wen Su:** Methodology (equal). **Shuo‐Chueh Chen:** Methodology (equal). **Liang‐Chih Liu:** Writing – original draft (equal). **Shun‐Fa Yang:** Conceptualization (equal); writing – original draft (equal); writing – review and editing (equal). **Yung‐Luen Yu:** Conceptualization (equal); writing – original draft (equal); writing – review and editing (equal).

## FUNDING INFORMATION

This research was funded by grants from the National Science and Technology Council, Taiwan (NSTC 109‐2320‐B‐039‐013‐MY3, NSTC 110‐2314‐B‐039‐048, NSTC 111‐2314‐B‐039‐070, NSTC 110‐2314‐B‐039‐034‐MY3, NSTC 112‐2320‐B‐039‐020), the National Health Research Institute, Taiwan (NHRI‐112A1‐CACO‐13222202), the China Medical University, Taiwan (CMU108‐MF‐01, CMU109‐MF‐03), the China Medical University Hospital, Taiwan (DMR‐111‐053, DMR‐112‐024, DMR‐112‐198).

## CONFLICT OF INTEREST STATEMENT

The authors declare no conflict of interest.

## INFORMED CONSENT

Informed consent was obtained from all subjects involved in the study.

## Data Availability

The data presented in this study are available on request from the corresponding author.
